# Maternal Perception of Reduced Fetal Movements Is Associated with Altered Placental Structure and Function

**DOI:** 10.1371/journal.pone.0034851

**Published:** 2012-04-16

**Authors:** Lynne K. Warrander, Gauri Batra, Giovanna Bernatavicius, Susan L. Greenwood, Philip Dutton, Rebecca L. Jones, Colin P. Sibley, Alexander E. P. Heazell

**Affiliations:** 1 Maternal and Fetal Health Research Centre, School of Biomedicine, Manchester Academic Health Science Centre, University of Manchester, Manchester, United Kingdom; 2 Department of Paediatric Histopathology, Royal Manchester Children's Hospital, Manchester, United Kingdom; 3 St Mary's Hospital, Central Manchester University Hospitals NHS Foundation Trust, Manchester, United Kingdom; Université de Montréal, Canada

## Abstract

**Background:**

Maternal perception of reduced fetal movement (RFM) is associated with increased risk of stillbirth and fetal growth restriction (FGR). DFM is thought to represent fetal compensation to conserve energy due to insufficient oxygen and nutrient transfer resulting from placental insufficiency. To date there have been no studies of placental structure in cases of DFM.

**Objective:**

To determine whether maternal perception of reduced fetal movements (RFM) is associated with abnormalities in placental structure and function.

**Design:**

Placentas were collected from women with RFM after 28 weeks gestation if delivery occurred within 1 week. Women with normal movements served as a control group. Placentas were weighed and photographs taken. Microscopic structure was evaluated by immunohistochemical staining and image analysis. System A amino acid transporter activity was measured as a marker of placental function.

Placentas from all pregnancies with RFM (irrespective of outcome) had greater area with signs of infarction (3.5% vs. 0.6%; p<0.01), a higher density of syncytial knots (p<0.001) and greater proliferation index (p<0.01). Villous vascularity (p<0.001), trophoblast area (p<0.01) and system A activity (p<0.01) were decreased in placentas from RFM compared to controls irrespective of outcome of pregnancy.

**Conclusions:**

This study provides evidence of abnormal placental morphology and function in women with RFM and supports the proposition of a causal association between placental insufficiency and RFM. This suggests that women presenting with RFM require further investigation to identify those with placental insufficiency.

## Introduction

There has been little reduction in stillbirth rates in high-income countries over the past 20 years, with the incidence of stillbirth in the UK and the USA approximately ∼5–6 per 1,000 live births [1], [2]. This results in part from a lack of sufficiently sensitive and specific methods of identifying women at highest-risk of stillbirth [3]. One clinical sign related to stillbirth is a reduction in maternally-perceived fetal movements. The majority of stillbirths are preceded by a period of reduced fetal movements (RFM) for 3–4 days and stillbirth rates are increased in women after RFM [4], [5], [6], [7]. However, the use of RFM as a screening tool to identify women at increased risk of stillbirth is contentious; this is in part related to the lack of pathophysiological evidence linking RFM to stillbirth.

Both pathological and non-pathological conditions are associated with RFM [8]; the most common pathological association with RFM is small for gestational age (SGA) or fetal growth restriction (FGR), which affects approximately 20% of pregnancies with RFM [9], [10], [11]. The link between RFM, SGA/FGR and stillbirth is proposed to be via placental insufficiency, where impaired placental function leads to oxygen and nutrient deprivation resulting in fetal compensation and impaired fetal growth. Ongoing placental insufficiency leads to RFM and ultimately fetal decompensation and death [12]. This hypothesis is supported by observations of relative hypoxaemia and acidaemia in fetuses after RFM compared to normal movements [13].

In FGR/SGA the placenta exhibits features of abnormal villous structure, reduced vascularity [14], [15], aberrant cell turnover [16], [17], [18], [19], as well as a reduced capacity for nutrient exchange [20], [21]. There are no similar studies investigating these indices of placental cell turnover and function in cases of stillbirth, decreased villous vascularity is evident in stillbirth with chronic fetal vascular insufficiency [22]. However, no study to date has investigated whether there is evidence of abnormal placental structure or function in RFM. The purpose of this study was to test the hypothesis that placentas from women who had presented with RFM in the week before delivery would have placentas with abnormal morphology and function.

## Methods

### Placental collection and Tissue Processing

Ethical approval was obtained from Oldham and North West Research Ethics Committees (Refs 08/H1011/83 and 08/H1010/55) and all participants provided written informed consent.

Tissue classified as normal was collected from women with uncomplicated pregnancies delivering at term with no history of RFM (n = 36). All women at St Mary's Hospital receive standard information recommending they present to the maternity service if they perceive a reduction in their babies' movements. From August 2009 until October 2010 women presenting with RFM after 28 weeks gestation were recruited from the Maternity Day Unit (n = 305). RFM was defined as subjective maternal perception of RFM for at least 12 hours [23]. Women were excluded if there was a fetal anomaly, multiple pregnancy or abnormal fetal heart rate on cardiotocography. If participants with RFM delivered within 7 days of presentation (n = 36), their placenta was collected. This seven day cut-off was arrived at empirically to allow a temporal association between RFM and placental pathology. For all women recruited the pregnancy outcome was recorded. Poor pregnancy outcome was defined as stillbirth, pre-term birth, small for gestational age infant defined as an individualised birthweight centile <10^th^ or term admission to the neonatal intensive care unit. These were chosen as infants stillborn after RFM were all SGA [24], and infants subject to severe intra-uterine compromise might not die but instead be delivered prematurely or require neonatal intensive care [25].

Placentas were collected from RFM and normal pregnancies within 30 minutes of birth. Placentas from either group were collected during the same time period and an identical sampling strategy used. Photographs of the maternal and fetal sides of the placenta were taken and the placenta was weighed, before and after removal of the membranes and cord. Biopsies of villous tissue (approximately 1 cm^3^) were dissected from the centre and edge of the placenta and a point midway in between. Tissue was fixed in 4% neutral buffered formalin for 24 hours and wax embedded. To analyse activity of the System A amino acid transport system, three 1 cm^3^ samples of villous tissue were randomly taken and placed in 1∶1 Dulbecco's Modified Eagle Medium (DMEM): control Tyrode's buffer [135.0 mM/L NaCl, 5.0 mM/L KCl, 1.8 mM/L anhydrous CaCl_2_, 1.0 mM/L MgCl_2_ hexahydrate, 10.0 mM/L HEPES, 5.6 mM/L D-glucose (pH 7.4)] for immediate analysis. Unless otherwise stated, reagents were acquired from Sigma-Aldrich (Poole, UK).

### Immunohistochemistry

Placental cell turnover, structure and vascularity were assessed using antibodies specific for Ki67 (Dako, Ely, Cambridgeshire, UK; 0.16 µg/ml), cytokeratin-M30 (Roche, London, UK; 0.66 µg/ml), cytokeratin 7 (Dako; 0.9 µg/ml) and CD31 (Dako; 0.16 µg/ml). Negative controls were performed using non-immune mouse IgG (Dako) at matching concentrations to the primary antibody.

Slides were dewaxed and antigen retrieval performed by microwaving the sections for 10 minutes in 0.01 M sodium citrate buffer. Endogenous peroxidase was quenched with 3% H_2_O_2_ before non-immune block (NIB, 10% goat serum, 2% human serum, in TBS-Tween 0.1% (Biorad Laboratories Ltd, Hemel Hempstead, Hertfordshire, UK) was applied for 30 minutes. Primary antibody/negative control diluted in NIB was incubated overnight at 4°C. Sections were then exposed to the biotinylated-goat anti-mouse IgG (4 µg/ml) for 30 minutes, followed by avidin peroxidase (5 µg/ml) for 30 minutes. Positive antibody binding was revealed by application of 3,3′-diaminobenzidine. Slides were counterstained, dehydrated and cleared before coverslips were applied with DPX (Raymond Lamb, London, UK).

Haematoxylin and eosin staining was performed to allow quantification of syncytial knots. Dewaxed and rehydrated sections were stained with Harris's haematoxylin for 10 minutes before differentiation in acid-alcohol. Slides were stained with eosin for 2 minutes, rinsed in cold tap water, and dehydrated and mounted as described above.

Images were captured using an Olympus BX41 light microscope (Southend-on-Sea, UK) and QIcam Fast 1394 (QImaging, BC, Canada) and Image Pro Plus 6.0 (Media Cybernetics Inc, MD, USA). For every section, 10 random images of terminal villi were taken, giving a total of 30 images per placenta for each component evaluated.

### Assessment of Placental Structure

Macroscopic placental structure was assessed using Image ProPlus 6.0 Software. The surface area, maximum, minimum and mean diameter, and percentage of placenta are with abnormal pale appearances were measured from photographs using the ‘area of interest’ tool; these were also confirmed to be pale on the cut surfaces of the placenta. A measure of placental roundness was calculated and cord insertions were determined through analysis of the photographs and classified using an established system, as follows: central, eccentric, marginal or velamentous [26].

Microscopic structure was evaluated as follows: the number of syncytial knots were counted and total villous area measured using image analysis software (Image ProPlus 6.0), expressed as the number of syncytial knots per mm^2^ of villous tissue as previously described [19]. Proliferative index was the number of Ki67 positive nuclei as a proportion of total nuclei as previously described [27]. Apoptosis was assessed by the number of nuclei surrounded by cytoplasmic M30 staining as proportion of total nuclei in the image. Vascularity was expressed as the number of capillaries per terminal villus. Trophoblast area was expressed as the proportion of villous area positive for CK-7 immunostaining.

In addition to quantitative analysis placental tissue stained with haematoxylin and eosin was assessed by a perinatal histopathologist (GB) who was blinded to the outcome of pregnancy. For each placenta three samples were analysed, and were qualitatively assessed for the presence of infarction, excessive syncytial knots, distal villous hypoplasia, villous immaturity, villitis and other incidental abnormalities.

### Placental System A uptake activity

Placental villous fragments (∼3–4 mm^3^) were incubated in a solution of radiolabelled-N-methylated aminoisobutyric acid (^14^C-MeAIB) (0.5 µCi/ml; 8.5 µM) (Perkin-Elmer, Cambridge, Cambridgeshire) with either control Tyrode's or Na^+^-free Tyrode's [NaCl replaced with 135.0 mM/L choline chloride] buffer for 30, 60 or 90 minutes at 37°C as previously described [28]. After incubation, the explants were lysed in dH_2_0 for 16–18 hours at room temperature. The radioactive content of the water was measured using a β-scintillation counter. The protein content of each villous fragments was determined by placing the tissue in 0.3 M NaOH for a minimum of 6 hours at 37°C, then using the Bio-Rad protein assay (Bio-Rad Laboratories Ltd). Radioactive counts were corrected for protein content. As System A is Na^+^-dependent, its activity was calculated by subtracting uptake in Na^+^-free Tyrode's from that in control Na^+^-containing Tyrode's.

### Statistical analysis

Power calculations were performed using PS Power and Sample Size Calculation (Version 3.0, January 2009). Power calculations were based on our previous data on syncytial knot density [19] and apoptosis [29] as data were not available for other indices. For a 90% power and an alpha value of 0.05, 6 experimental samples would be required to show a significant difference in the number of syncytial knots and 12 experimental samples would be required to show a significant difference in apoptosis between control pregnancies and RFM ending in a poor pregnancy outcome. As we anticipated that between 25–33% of our placental samples would have a poor pregnancy outcome, 24–36 subjects with RFM would be required for an adequately powered study.

Statistical analysis was performed using GraphPad Prism 4.0 (GraphPad Software, La Jolla, CA, USA) and Joosse Online Statistics http://in-silico.net/statistics/fisher_exact_test. Normality of the data was first tested using the Kolmogorov-Smirnov test. For normally distributed continuous data, Student's t test was used, and for non-normally-distributed continuous data Mann Whitney U Test was used. For analysis of more than 2 data sets Kruskal-Wallis with Dunn's post-hoc test was used (non-parametric). Categorical data were tested using Fishers' exact test. System A activity was assessed by two-way ANOVA.

## Results

### Demographic data

During the study period 7,651 women gave birth at St Mary's Hospital. Of these 351 presented to the maternity day unit with RFM, of whom 305 agreed to participate in the study. 36 participants gave birth within 7 days of assessment. Table 1 shows the demographic data for women with RFM and control pregnancies from whom placental tissue was collected. There was no significant difference in other maternal demographic details, gestation, or induction of labour. Women in the control group were more likely to have had elective Caesarean section.

**Table 1:** Demographic details of subjects with RFM.

		RFM (n = 36)	Normal (n = 36)	P value
Maternal age		27 yrs (17–41)	30 yrs (18–41)	0.10
Gravidity		2 (1–8)	2 (1–7)	0.86
Parity		1 (0–4)	1 (0–6)	0.55
Body Mass Index		24.4 (18.1–45.6)	24.6 (17.8–41.9)	0.88
Gestation at delivery		40^+1^ (30^+1^–42^+0^)	40^+1^ (37^+4^–42^+2^)	0.18
Cigarette Smoking		8 (22.2%)	5 (13.9%)	0.54†
Pre-existing complications in Pregnancy*		4 (11.1%)	0 (0%)	0.11†
Evidence of fetal	Estimated fetal weight <10^th^ centile	8# (22.2%)	-	
compromise at	Abnormal umbilical artery Doppler	1# (2.7%)	-	
presentation with RFM	Abnormal cardiotocography trace	1# (2.7%)	-	
Recurrent presentation with RFM		7 (19.4%)	-	
Induction of Labour		10 (27.7%)	4 (11.1%)	0.08†
Caesarean Section		9 (25.0%)	21 (58.3%)	0.008†
Birthweight centile		15.3 (0–100)	58.5 (12–98)	P<0.0001†***
Poor pregnancy outcome**		12 (33.0%)	0 (0%)	p<0.005† **
Admission to NICU		3 (8.3%)	1 (2.8%)	1.00†

Continuous data are presented as medians with range in parentheses.

# One participant had estimated fetal weight <10^th^ centile, abnormal umbilical artery Doppler and abnormal fetal heart rate trace.

* Complications in pregnancy included oligohydramnios and gestational diabetes.

** Poor pregnancy outcome defined as birthweight <10^th^ centile, delivery before 37 weeks gestation or admission to the neonatal intensive care unit (NICU).

† Differences between proportions tested using Fishers' exact test.

Twelve women (33%) with RFM had a poor pregnancy outcome, 8 were SGA at term, 3 were SGA born preterm, and one was preterm with a normal birthweight. The birthweight centile is skewed towards the lower values in women with RFM (RFM median 15 (range 0–100) compared to control 51 (12–96)), reinforcing the association between RFM and SGA. Six of the 12 patients with poor outcome after RFM had evidence of placental pathology at the time of initial assessment (oligohydramnios, abnormal umbilical artery Doppler). All participants with normal outcome after RFM had normal estimated fetal weight, liquor volume and umbilical artery Doppler at initial assessment.

### Placental structure

**Figure 1:**
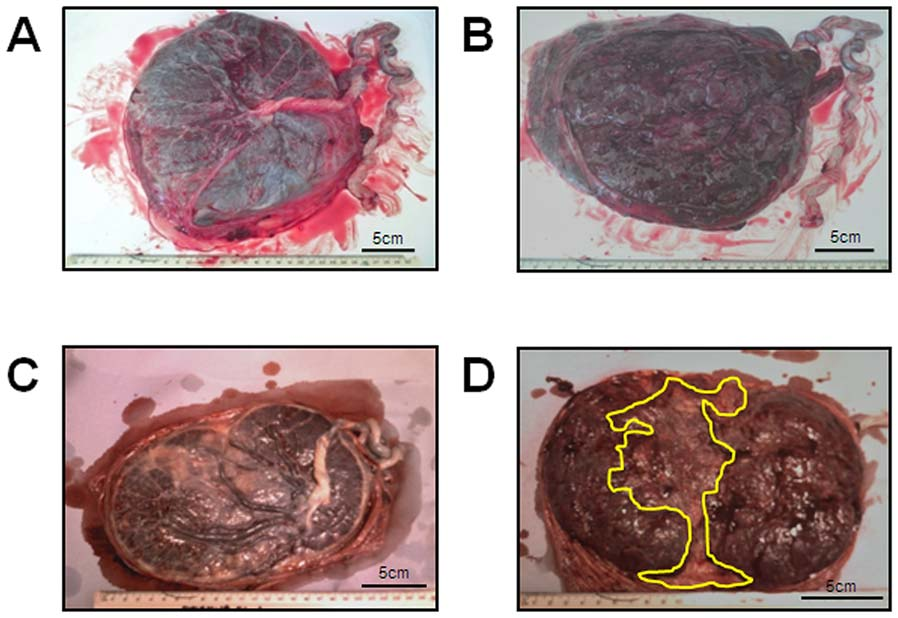
Examples of placental photographs from uncomplicated (A–B) and RFM (C–D) pregnancies. **A**) Fetal side of a control placenta with a central cord insertion and normal distribution of chorionic plate vessels. **B**) Shows the maternal side of a control placenta. **C**) Shows the fetal side of a RFM placenta which is less round than the control example, and the cord is inserting laterally. **D**) Shows the maternal side of a RFM placenta. The area highlighted by the yellow line shows an abnormal white area which was confirmed on the cut surface of the placenta.

Macroscopic changes in placental structure were evident in RFM compared to control pregnancies (Table 2). In comparison to normal pregnancies, placentas from RFM are lighter, have a smaller surface area, a higher fetal-placental weight ratio and a greater proportion taken up with abnormal appearing white areas (Figure 1) which were also visible on cut surface of the placenta. They are also more likely to have a non-central cord insertion. To evaluate whether this effect was primarily due to pregnancies with poor pregnancy outcome, a further analysis was performed comparing normal pregnancies to RFM with a normal outcome and to RFM with a poor outcome. This demonstrated that only placentas from RFM with a poor outcome had a smaller placental weight and size than normal controls. However, the fetal∶placental weight ratio, the proportion of placenta with abnormal white areas and the number of placentas with non-central cord insertion differed between normal controls and RFM irrespective of the outcome of pregnancy.

**Table 2:** Macroscopic features in placentas from uncomplicated pregnancies and those complicated by RFM.

	Normal	RFM Total	RFM Normal Outcome	RFM Poor Outcome	*Normal vs. RFM Total*	*Normal vs. RFM Poor Outcome*	*Normal vs. RFM Normal Outcome*
Untrimmed placental weight (g)Δ	657.9±20.1	587.6±26.9	634.2±28.4	503.1±48.4	p = 0.03*	p = 0.0009**	*p = 0.42*
Trimmed placental weight (g)Δ	544.9±17.3	469.2±22.7	513.2±24.0	383.8±36.4	p = 0.01**	p<0.0001***	*p = 0.27*
Placental weight centile							
<3^rd^	0	4	1	3	*p = 0.065*	p = 0.011*	*p = 0.281*
4^th^–9^th^	3	4	1	3			
10–49^th^	19	21	16	5			
50–89^th^	11	6	5	1			
90–96^th^	3	0	0	0			
>97^th^	0	1	1	0			
Fetal/Placental Weight Ratio+	5.69 (5.25–6.02)	6.76 (5.69–7.64)	6.73 (5.70–7.70)	6.56 (5.51–7.03)	p<0.0001***	*p = 0.07*	p = 0.0002***
Placental surface area (cm^2^)Δ	253.2±8.7	222.3±9.1	236.4±10.2	181.5±12.7	p = 0.01**	p = 0.0004***	*p = 0.22*
Mean diameter (cm)Δ	17.76±0.31	16.59±0.35	17.12±0.38	15.04±0.54	p = 0.01**	p = 0.0002***	*p = 0.19*
RoundnessΔ	0.28±0.03	0.29±0.03	0.28±0.03	0.34±0.03	*p = 0.70*	*p = 0.96*	*p = 0.39*
% of maternal surface abnormal pale area+	0.49 (0–1.21)	2.57 (0.39–4.10)	1.24 (0–3.86)	4.07 (2.58–15.5)	p = 0.003**	p<0.0001***	p = 0.05*
Cord insertion†							
Central	18 (50%)	10 (28%)	7 (29%)	3 (25%)	p<0.0001***	p = 0.0008***	p = 0.0008***
Eccentric	14 (38%)	14 (38%)	10 (42%)	4 (33%)			
Marginal	3 (6%)	12 (28%)	6 (25%)	4 (33%)			
Velamentous	2 (6%)	2 (6%)	1 (4%)	1 (9%)			

Δ = Unpaired t test (Data shown are Mean ± SEM),

+ = Mann Whitney U Test (Data shown are Median (IQR)),

† = Fishers' Exact Test. Poor outcome = SGA (individualised birthweight centile ≤10), preterm birth ≤37 weeks, admission to NICU.

Qualitative histopathological examination showed that samples from RFM more often had excessive syncytial knots (p<0.001) and infarction (p<0.01) (Table 3). All samples from RFM showed excessive syncytial in at least one area of the placenta. The areas affected by infarction showed early changes of ischaemia. Other abnormalities identified in RFM included: chronic villitis (focal), patchy hydropic changes and chorangiosis.

**Table 3:** Classification of samples on histopathological review.

Histological Feature	Normal (n = 18)			Reduced Fetal Movements (n = 36)			p value
	No samples	1–2 samples	All samples	No samples	1–2 samples	All samples	
Infarction	18 (100)	0 (0)	0 (0)	25 (69)	11 (31)	0 (0)	0.01
Excessive Syncytial Knots	8 (45)	6 (33)	4 (22)	0 (0)	10 (28)	26 (72)	<0.0001
Distal Villous Hypoplasia	11 (61)	5 (28)	2 (11)	13 (36)	18 (50)	5 (14)	0.24
Villous Immaturity	16 (89)	2 (11)	0 (0)	31 (86)	2 (6)	3 (8)	0.56
Villitis	18 (100)	0 (0)	0 (0)	34 (94)	2 (6)	0 (0)	0.11
Other	18 (100)	0 (0)	0 (0)	32 (89)	4* (11)	0 (0)	0.28

Statistical significance tested using Fisher's exact test.

* = 2 patchy hydropic villi and 2 chorangiosis. % of total samples is shown in brackets for each feature assessed.

**Figure 2:**
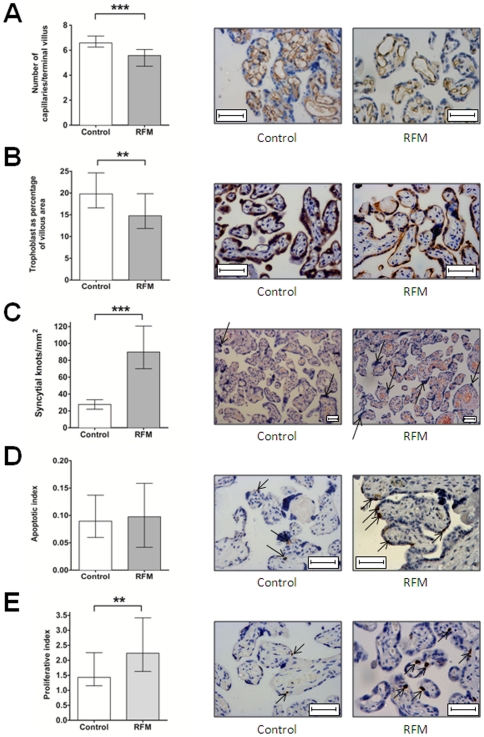
Microscopic structure of RFM placentas compared to control pregnancies. A) RFM placentas have significantly decreased villous vascularity (*** p<0.001, Mann-Whitney U test). Representative micrographs of CD31 immunostaining. B) In RFM, the placenta has significantly reduced trophoblast area compared to control placentas (**p<0.01, Mann Whitney U Test). Representative micrographs of CK7 immunostaining. C) Density of syncytial knots is increased in RFM (*** p<0.001, Mann-Whitney U Test). Representative images from haematoxylin and eosin staining, syncytial knots are marked with arrows. D) There is no significant difference in cell death through apoptosis in RFM compared to control placentas. Representative images from M30 staining; M30 staining is marked with arrows. E) Proliferative Index is increased in RFM (**p<0.01, Mann-Whitney U Test) Representative images from Ki67 immunostaining (Mib1 antigen). Positive nuclei are marked with arrows. In all photomicrographs bar = 50 µm.

**Figure 3:**
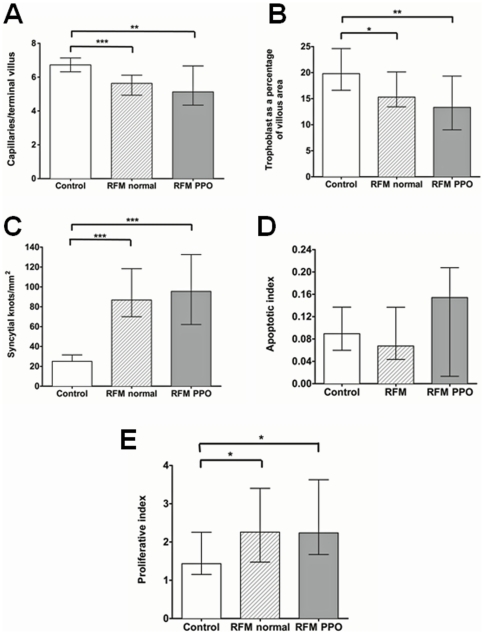
Microscopic structure of RFM placentas split by those with normal and poor pregnancy outcomes (PPO). demonstrating that the same pattern in abnormal villous structure is present in all women with RFM with respect to: A) Villous vascularity B) Trophoblast area C) Density of syncytial knots D) Apoptosis E) Proliferative Index. (* p<0.05, **p<0.01, ***p<0.001, Kruskal-Wallis Test).

When quantifying changes in microstructure, there was significantly reduced villous vascularity and syncytiotrophoblast area in RFM but increased density of syncytial knots (Figure 2 A–C) and proliferative index (Figure 2E). There was no difference in rates of apoptotic cell death as assessed by M30 immunostaining (Figure 2D). When the RFM group was divided according to pregnancy outcome significant differences in microscopic placental structure were observed between normal control pregnancies and RFM with both normal and poor pregnancy outcome (Figure 3).

A correlation between cases with abnormal CTG, oligohydramnios or abnormal umbilical artery Doppler and the microscopic analyses in placentas from pregnancies with RFM is not possible due to the small numbers of these abnormalities. However, the relationship between birthweight centile and the microscopic indices was assessed. There was no significant correlation between birthweight centile and the density of syncytial knots, apoptotic index, proliferative index or the number of vessels per villus. There was a positive correlation between trophoblast area (r^2^ = 0.12, p = 0.04) and placental weight (r^2^ = 0.32, p = 0.003) with birthweight centile.

### System A amino acid transporter activity

**Figure 4:**
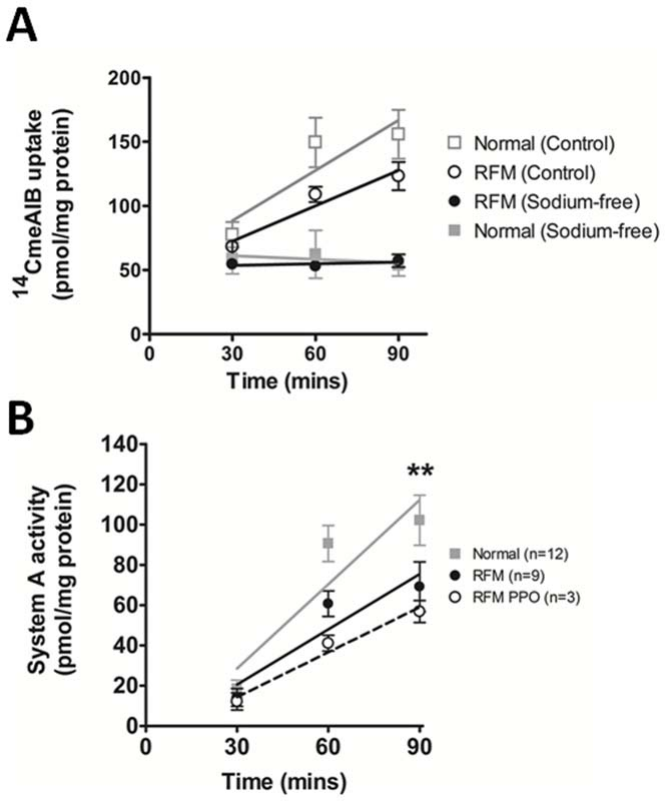
System A amino acid transporter activity. A) System A transporter activity is sodium-dependent, as no uptake is seen in either normal of RFM placental fragments in the absence of sodium (solid boxes). Uptake is evident in fragments incubated in the presence of sodium (hollow boxes). B) RFM placentas have significantly lower system A activity than normal pregnancies. The RFM group with poor pregnancy outcome (PPO) have the lowest system A activity. [Mean ± SEM; ** = P<0.01, Two-way ANOVA].

The activity of the system A amino acid transporter was analysed in 12 placentas from pregnancies associated with RFM and 12 placentas from uncomplicated pregnancies (Figure 4). In the presence of Na^+^^14^CMeAIB uptake increases over time in both control and RFM placentas (Figure 4A) and uptake is higher than in the absence of Na^+^. The Na^+^-dependent component of ^14^CMeAIB uptake (System A activity) is significantly reduced in placental villous explants from pregnancies associated with RFM, when compared to samples taken from uncomplicated pregnancies (Figure 4B). The reduction in system A activity was even more pronounced in women with RFM associated with a poor pregnancy outcome (n = 3) (Figure 4B).

## Discussion

Placentas from women who reported RFM within a week of delivery had significant differences in placental structure and function when compared to placentas taken from uncomplicated pregnancies with normal movements. These changes in the structure and function of the placenta are similar to the hallmarks of placental insufficiency in SGA [19], [30], [31]. For example, the increase early placental changes of infarction was reminiscent of changes present in stillbirths with SGA [32]. Abnormalities in cord insertion were also evident in RFM and are similar to observations made by Biswas and Ghosh in SGA pregnancies [26]. This combination of lateral cord insertion and abnormal placental shape has been proposed by Salafia et al. to lead to reduced placental efficiency, possibly due to alterations of placental vascularity and branching [33], [34], providing a mechanism linking abnormal placental shape with RFM, SGA and stillbirth.

There have been no studies of apoptosis, proliferation and trophoblast area in stillbirths to enable comparison with the data from RFM. However, RFM was associated with several abnormalities of placental villous architecture including reduced villous vascularity and reduced area of the trophoblast in the terminal villi. Reduced villous vascularity has been previously found in SGA and stillbirth [14], [32]. Likewise, syncytiotrophoblast area has been correlated with birthweight and is reduced in SGA [30]. The combination of these anomalies will reduce the functional capacity of the placenta thereby decreasing the supply of oxygen and nutrients to the fetus, resulting in SGA. The density of syncytial knots was greatly increased in RFM compared to normal placental samples which agrees with previous work that has shown increased syncytial knots in placental pathologies such as SGA and pre-eclampsia [35], [36]. These quantitative changes in syncytial knots were present in placentas from RFM irrespective of the pregnancy outcome and were confirmed by histopathological assessment of the placenta. Taken together the macroscopic and microscopic changes suggest that there is a degree of abnormal placental structure associated with RFM per se.

The abnormalities in villous structure are coupled with altered placental function in RFM. The activity of the System A amino acid transporter has been consistently shown to be reduced in placentas from women suffering SGA or FGR [21], [31] and was used here as a marker of placental nutrient transport function. The reduction in System A activity in placentas from women reporting RFM suggests that there is a degree of functional impairment in these placentas, alongside the structural abnormalities. Future studies will need to examine other aspects of placental function that might be altered in women reporting RFM.

As with all studies, this study has some limitations. Firstly, there is a potential for selection bias, if women presenting with RFM were only from high-risk pregnancies that had been told to monitor fetal movements closely, then this might bias the sample towards those with placental pathology. In fact, only 4 cases (11%) of RFM had pre-existing maternal or fetal complications making this possibility unlikely. Secondly, placental tissue was only collected from women who delivered within one week of presenting with RFM. This may have favoured collection of tissue from pregnancies which were induced or delivered for complications. However, to have obtained all placental tissue would make it difficult to link RFM in the early third trimester with placenta pathology evident at delivery at term. Thirdly, the inclusion of preterm delivery preterm may have altered the results as placental pathology may be different in preterm and term SGA. In total, 4 (11%) cases of RFM were preterm, and even when these cases are excluded the differences in placental microstructure are still evident. The mode of delivery may have affected the aspects of placental cell turnover and system-A transport. However, in these experiments, analysis of samples from women who had Caesarean section versus Vaginal Delivery showed no difference in microscopic placental structure or system A activity.

None of the women recruited to the study or this placenta cohort had pregnancies resulting in stillbirth. This may in part be due to participation in a clinical study (the Hawthorne effect) or the improved standardised care that all women recruited onto the study received so that signs of fetal compromise were managed appropriately. Thus, structural changes in the placenta in stillbirth after RFM have not been investigated. Through subdividing the RFM group into those with normal outcome and those with poor pregnancy outcome it has been possible to show that the abnormalities seen cannot solely be attributed to those placentas associated with overt pathology, where structural abnormalities are well documented. This provides evidence to confirm the study hypothesis, and to suggest that in some women RFM is associated with a degree of placental insufficiency.

These data linking RFM with changes to placental structure and impaired amino acid transport suggest that women presenting with RFM should be taken seriously and assessed carefully to identify evidence of placental insufficiency; specifically acute fetal compromise or FGR/SGA. Current tests to identify placental insufficiency include clinical assessment of fetal size by measurement of symphysiofundal height through to ultrasound biophysical profiles but strategies are used inconsistently [25], [37], [38]. Currently, there is little evidence to demonstrate which test (if any) has sufficient sensitivity or specificity to identify babies at the highest risk of stillbirth to direct intervention [25]. Given the evidence of placental insufficiency in some women with RFM, a placental biomarker may be a useful predictor of poor pregnancy outcome in this group. A study is underway to measure such placental biomarkers and determine their efficacy in predicting poor pregnancy outcome in a cohort of women presenting with RFM.
